# Angiogenesis: Managing the Culprits behind Tumorigenesis and Metastasis

**DOI:** 10.3390/medicina54010008

**Published:** 2018-03-25

**Authors:** Ashwaq Hamid Salem Yehya, Muhammad Asif, Sven Hans Petersen, Ayappa V. Subramaniam, Koji Kono, Amin Malik Shah Abdul Majid, Chern Ein Oon

**Affiliations:** 1 Institute for Research in Molecular Medicine (INFORMM), Universiti Sains Malaysia, Penang 11800, Malaysia; ashwaqlabwork@gmail.com (A.H.S.Y.); ayappa725@gmail.com (A.V.S.); 2 Faculty of Pharmaceutical Sciences, Government College University, Faisalabad 38000, Pakistan; drmasif@gcuf.edu.pk; 3 Cancer Science Institute of Singapore, National University of Singapore, Singapore 117543, Singapore; svenhanspetersen@tessatherapeutics.com (S.H.P.); kojikono@fmu.ac.jp (K.K.); 4 Department of Surgery, National University of Singapore, Singapore 117543, Singapore; 5 School of Medicine, Fukushima Medical University, Fukushima 960-1295, Japan; 6 EMAN Testing and Research Laboratories, Department of Pharmacology, School of Pharmaceutical Sciences, Universiti Sains Malaysia, Penang 11800, Malaysia; aminmalikshah@gmail.com; 7 ACRF Department of Cancer Biology and Therapeutics, The John Curtin School of Medical Research, Australian National University, Acton 0200, Australia

**Keywords:** angiogenesis, growth factor, endothelial cells, chemotherapy resistance, complimentary combination

## Abstract

Deregulated angiogenesis has been identified as a key contributor in a number of pathological conditions including cancer. It is a complex process, which involves highly regulated interaction of multiple signalling molecules. The pro-angiogenic signalling molecule, vascular endothelial growth factor (VEGF) and its cognate receptor 2 (VEGFR-2), which is often highly expressed in majority of human cancers, plays a central role in tumour angiogenesis. Owing to the importance of tumour vasculature in carcinogenesis, tumour blood vessels have emerged as an excellent therapeutic target. The anti-angiogenic therapies have been shown to arrest growth of solid tumours through multiple mechanisms, halting the expansion of tumour vasculature and transient normalization of tumour vasculature which help in the improvement of blood flow resulting in more uniform delivery of cytotoxic agents to the core of tumour mass. This also helps in reduction of hypoxia and interstitial pressure leading to reduced chemotherapy resistance and more uniform delivery of cytotoxic agents at the targeted site. Thus, complimentary combination of different agents that target multiple molecules in the angiogenic cascade may optimize inhibition of angiogenesis and improve clinical benefit in the cancer patients. This review provides an update on the current trend in exploitation of angiogenesis pathways as a strategy in the treatment of cancer.

## 1. Introduction

**Figure 1:**
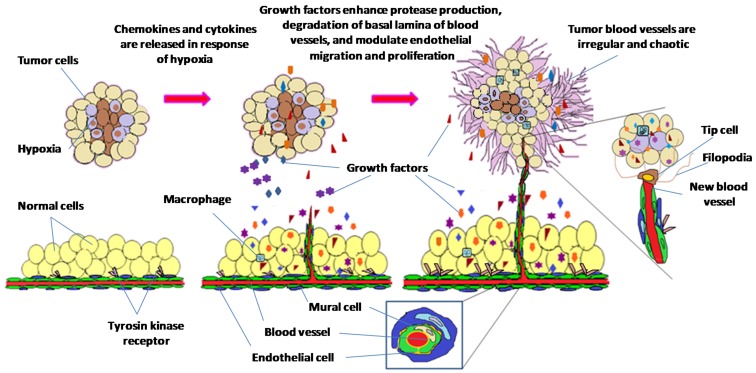
Progression in tumor angiogenesis. Hypoxic tumor microenvironment triggers cells to produce and release chemokines and cytokines. The overexpression of growth factors enhances protease production leading to degradation of vessel basal lamina, and modulates endothelial cell migration and proliferation. Tip cell guides the sprouts of new blood vessels towards the stimulus. Recruitment of mural cells and generation of new basal membrane enhance blood vessel maturation.

Angiogenesis, the growth of new blood vessels, is central to tumor growth and metastasis [1]. Matrix degradation, endothelial cell proliferation, migration, sprouting and recruitment of mural cells takes place during this process [2]. The growth of new blood vessels depends on the balance between pro-angiogenic and anti-angiogenic factors, during which angiogenic switch gets activated when pro-angiogenic stimulus is stronger than anti-angiogenic resistance. Hypoxic tumor microenvironment triggers release of growth factors which stimulate vascular endothelial cells to sprout and migrate, which in turn causes release of proteases that enhance degradation of basal lamina of blood vessels. Sprouting subsequently creates profuse network of vessels that transport nutrients and oxygen to fuel tumor growth (Figure 1). Overexpression of angiogenic factors is often associated with hypervascular nature of tumor angiogenesis [3]. Morphology of tumor vessels and extracellular matrix proteins on cell surfaces are abnormal compared to normal vessels, hence many of these proteins are used as markers to distinguish between tumor blood vessels and normal blood vessels [4,5].

Angiogenesis being the main basis of tumor growth and metastasis has been a subject undergoing intense study. Targeting the proteins or mediators that are involved in promoting angiogenesis has thus provided a great platform for future therapeutic treatment of cancer. As such this article intends to explore the current trends in exploitation of angiogenesis pathways as a strategy in the treatment of cancer.

## 2. Targeting Tumor Vasculature as a Therapeutic Strategy

Linings of the entire vascular system including blood and lymphatic vessels are made up of endothelial cells (EC) [6,7]. In angiogenesis and lymphangiogenesis vascular and lymphatic ECs play a very important role as versatile and multifunctional organs [6]. Regulation of angiogenic and lymphangiogenic processes depends on the heterogenous behaviour of EC that exhibits complex and diverse functions in different microenvironment [8]. In fact, these processes involve plenty of molecular regulators and signaling pathways. In addition to vascular endothelial growth factor (VEGF), other angiogenic factors including fibroblast growth factor (FGF), human epidermal growth factor (HEGF), thromposondin 1 (TSP-1), endostatin and angiobiotin may act on ECs directly or indirectly by inducing expression of angiogenic factors [9].

Rapid growth of neoplastic cells in tumor mass together with overexpression of multiple pro-angiogenic factors often lead to development of vascular network that display numerous structural and functional abnormalities. Newly formed blood vessels usually display irregularity in functional perfusion alongside excessive branching and shunts [10,11]. Similarly, tumor vasculature lacks structural organization into arterioles, capillaries and venules and has uneven blood flow towards tumor mass. These structural abnormalities result in formation of hypoxic and acidic areas inside rapidly growing tumors [12]. In addition, a process known as vascular mimicry where tumor cells can be incorporated into endothelial wall followed by differentiation of tumor stem-like cells into ECs play an essential role in tumor vascular system [13]. Heterogeneous distribution of blood vessels is apparent with dense network of blood vessels which is present on invading tumor edges, while there is less blood supply towards the core of the tumor mass [13]. Furthermore, rapidly proliferating tumor cells can also exert pressure on tumor vasculature which results in generation of increased interstitial pressure [13,14]. This causes reduced blood supply and removal of metabolic wastes from tumor mass that leads to generation of hypoxic and acidosis condition within the tumor microenvironment. These structural abnormalities are responsible for impaired delivery of anticancer drugs to tumor mass as well as generation of chemotherapy and radiotherapy resistant clones [15,16]. Structural and functional abnormalities observed in tumor vasculature may result in dual effects. In light of this, many studies have looked into targeted delivery of therapeutic modalities especially when anti-angiogenic and chemotherapy drugs are combined together [17].

## 3. Molecular Mediators of Angiogenesis 

Tumor microenvironment (TME) is created by a complex and dynamic network of growth factors, cytokines, chemokines, inflammatory cells and matrix remodeling enzymes [18]. Individual role of these mediators in carcinogenesis is highlighted in the following sections.

### 3.1. Inflammatory Cells

Macrophages are innate immune cells differentiated from bone marrow-derived monocytic precursor cells [19]. Once these precursors arrive in their destined tissues, they are polarized into distinct macrophage subsets and display different phenotypes depending on the tissue microenvironment they reside in. Fundamentally, these subsets are comprised of the “classical M1” and the “alternative M2” macrophages via displaying specific expression profiles of cell-surface markers, enzymes and cytokines [20]. Characteristically, these cells produce pro-inflammatory cytokines such as Interleukin (IL)-6, -12, -23 and tumor necrosis factor (TNF) α. M1 and M2 macrophages counteract inflammation and perform reparative functions by contributing to wound healing, tissue repair and angiogenesis [20,21]. Macrophages are also found within stroma of tumors and are commonly referred to as tumor associated macrophages (TAMs) [22]. These macrophages support tumour growth and metastasis. Therefore, the development of therapeutic candidates to hinder the recruitment macrophages at primary and secondary tumour sites may be an important strategy to improve cancer survival [23].

### 3.2. Growth Factors

#### 3.2.1. Vascular Endothelial Growth Factors (VEGFs)

VEGFs are the most critical pro-angiogenic factors that enhance tumor growth and thus, have become an attractive target for angiogenesis therapy [24]. The VEGF family consists of seven ligands namely VEGF-A, -B, -C, -D, and -E, placenta growth factors (PIGFs)-1 and -2 [25]. The vascular endothelial growth factor receptors (VEGFR-1, -2, and -3) are basic transmembrane receptor tyrosine kinases that are able to form homodimers and heterodimers [26]. Dimerization of these receptors is accompanied by activation of receptor-kinase activity that leads to auto-phosphorylation of these receptors [27]. Cell migration, proliferation, survival, and mobilization of endothelial progenitor cells from the bone marrow into the peripheral circulation involves VEGFRs [24]. Moreover, these receptors have the ability to transduce signals within the vascular tubes to regulate vascular permeability that leads to oedema and swelling of tissues [25]. VEGFR-1 plays an important role in the physiological and developmental angiogenesis [27]. As the decoy receptor for VEGF-A, VEGFR-1 has the ability to bind to VEGF-B and PIGF [26]. VEGFR-1 works as a positive regulator of angiogenesis as well as macrophage and monocyte migration [25,28]. Besides that, the majority of the downstream effects in angiogenesis is mediated by VEGFR-2, another receptor for VEGF-A, which also mediates microvascular permeability, endothelial cell proliferation, migration, invasion and survival [26]. VEGFR-2 is regarded as the earliest marker for endothelial cell growth that directly controls the tumour angiogenesis. Autocrine/paracrine mechanisms in the processes of cancer cell survival and proliferation is mediated by the upregulation of VEGF/VEGFR-2 signalling [15,29]. VEGFR-3 on the other hand binds to VEGF-C and VEGF-D to enhance endothelial cell migration and proliferation [30]. Although VEGFR-3 is expressed in adult human with transient lymphangiogenesis and remodeling of primary vascular networks during embryogenesis, it is lowly expressed in the blood vessels during tumor angiogenesis [31]. VEGFR-3 is required for the initial steps of VEGFD-mediated lymphogenous metastasis, even though lymphogenous metastasis is reported to be less dependent on VEGFR-2 mediated angiogenesis [31]. Apatinib, axitinib, bevacizumab, and ramucirumab are antiangiogenic agents and targets VEGF and its receptors in different types of cancer [32,33,34].

#### 3.2.2. Fibroblast Growth Factors (FGFs)

FGFs comprise a family of nine related polypeptides that are mostly expressed in pituitary, brain, and eyes in mammals [35]. Fibroblast growth factor receptor (FGFR)-1, -2, -3, and -4, are structurally related four receptor tyrosine kinases which mediates the biological effects of FGF [36]. Acidic and basic FGFs have been well characterized as angiogenic factors which play an important role in cell proliferation, differentiation, and cell migration [37]. Acidic and basic FGFs have been reported to have a synergistic effect with VEGF and PDGF on microvascular endothelial cell proliferation model [37]. Furthermore, FGFs and their receptors (FGFRs) have been implicated in several human cancers growth and progression.FGFs are pleiotropic factors that exhibit paracrine and autocrine properties on tumor and stromal cells [38]. Thus, FGFs may represent key players in the complex crosstalk among tumor growth, angiogenesis, inflammation, and drug resistance that contribute in tumor progression [38]. Emibetuzumab is an antiangiogenic agent and targets FGFs in gastric cancer [39], whereas, lucitanib, pazopanib, and ponatinib are antiangiogenic agent and targets FGF receptors in different types of cancer [32].

#### 3.2.3. Platelet-Derived Growth Factors (PDGFs)

PDGFs are members of the growth factor family that binds to tyrosine kinase receptors α and β (PDGFR α and β) have been shown to play an important role during blood vessel development in both normal and pathological angiogenesis [40]. These factors stimulate fibroblast proliferation, survival, and migration to make contact withcollagen matrices and induce myofibroblast phenotypes in these cells [41]. PDGFs are also involved in growth factor-mediated integrin activation which is critical for cell proliferation and signalling in tumor angiogenesis [40,42]. In many cases, PDGF signalling can cooperate with integrin signalling to induce oligodendrocyte precursor proliferation via phosphotidylinositol-3 kinase (PI3K)-dependent signalling pathway [42]. PDGF signalling involves degradation of MAP-kinase phosphatase which enhances ERK-MAP-kinase activation in porcine aortic endothelial (PAE) and human embryonic kidney 293T cells, which further promotes cell migration, proliferation, and cell cycle progression [43]. PDGFRs have been reported to activate STAT transcription factors via activation of JAK kinases [44]. Cediranib, imatinib, lenvatinib, pazopanib, ponatinib, and sorafenib are used to target PDGFs in caner [32,45,46].

#### 3.2.4. Epidermal Growth Factors Receptors (EGFR) and Human Epidermal Growth Factors Receptor2 (HER2)

Emerging evidence has demonstrated importance of targeting angiogenesis using HER2 inhibitors [47]. EGFR and HER2 have been reported to mediate tumor angiogenesis by up-regulating VEGF and vascular permeability factors in cancer cells [48]. HER2 (c-erbB2) belongs to membrane tyrosine kinase family, which also includes HER1 (EGFR), HER3 (c-erbB3), and HER4 (c-erbB4) [48]. HER2 has been found to enhance corneal epithelial cell wound healing and neovascularization in rabbit models [47,49]. Vandetanib is an antiangiogenic agent and targets EGFR in medullary thyroid cancer [50].

#### 3.2.5. Transforming Growth Factor-B (TGF-B)

This family of growth factors is comprised of 30 members and three isoforms of TGF-β i.e., TGF-β 1–3. It is secreted by ECs and pericytes in an inactive form and needs cleavage by proteases in acidic environment and heat. Studies have shown that TGF-β acts as a pro-angiogenic and angiostatic agent. In vitro studies show that it acts as a anti-angiogenic agent in a receptor specific manner and down-regulates VEGFA expression through PKA-mediated pathway [51]. On the contrary, results from in vivo studies show that it modulates EC wound proliferation, migration and capillary tube formation owing to its ability to recruit inflammatory cells, which release pro-angiogenic molecules. It also modulates the activity of other angiogenic pathways which may account for its pro-angiogenic effects in vivo [52].

#### 3.2.6. Angiopoietins (Angs)

Angiopoietins represent family of extracellular ligands which bind with Tie receptors present on surface of ECs. There are four members in this family i.e., Angiopoietin 1, 2, 3 and 4. Angiopoietin (Ang) 1 and 2 bind with Tie-2, but elicit very different responses. Angiopoietin 1 (Ang 1), secreted by vascular smooth muscle and other periendothelial cells, lacks ability to induce ECs proliferation or tube formation in vitro, but it promotes sprouting of ECs. Binding of Ang-1 with its receptors, causes rapid receptor trans-phosphorylation, with subsequent activation of protein kinase B/Akt/FKHR (FOX01) downstream pathway which in turn is responsible for ECs survival [53]. Ang 2 has been shown to have a broad spectrum of effects (angiogenic as well as angiostatic) in angiogenic cascade depending on the type of co-stimulatory molecules present. During early angiogenic events in the presence of angiogenic stimuli (VEGF and hypoxia) it causes dramatic increase in the number of ECs by competing with Ang 1 to bind with Tie 2 receptors and prevents receptor auto-phosphorylation. Ang 2 promotes angiogenesis via EC survival, migration, capillary diameter expansion and differentiation into tubular network [13]. In vivo studies show that Ang 2 is highly expressed in vascularised tumors. In absence of angiogenic stimuli Ang 2 however acts as an antiangiogenic agent leading to induction of apoptosis in ECs and vessel regression [53].

### 3.3. Cytokines and Chemokines

Cytokines are proteins that are secreted by the innate and adaptive immune system to regulate the different biological functions in immune response [54]. The cytokines are structurally similar however they exist in broad families and perform different functions [54]. Chemokine superfamily has a wide rangeof lowmolecular weight chemotactic proteins that are involved in regulation of multiple steps of tumorprogression and metastasis including proliferation, neovascularization, invasion and migration of malignant cells to distinct organs [55]. This family is divided into two broad categories mainly thosehaving “ELR” motif (ELR^+^ or ELR_1_) which are potent promoters of angiogenesis, whereas members that are induced by interferon and lack ELR motif (ELR^−^ or ELR_2_) which are potent angiogenic inhibitors with exception of chemokine (C-X-C motif) ligand (CXCL12) [56]. CXCL8 is one of most extensively studied chemokines in the ELR^+^ category, as a potent angiogenic mediator in a variety of in vitroand in vivo assays [57]. In vitro studies have shown that CXCL8 receptors (CXCR1 and CXCR2) are located on surface of epithelial cells, CXCL8 binds with these and induces ECs proliferation, and differentiation into a capillary-like networkand inhibits apoptosisin a dose dependent manner [58]. CXCL8 exerts its angiogenic activity by up-regulating matrix metalloproteinase (MMP-2) and MMP-9 enzymes in tumor and endothelial cells leading to degradation of extracellular matrix which is one of pre-requisites for EC migration and organization [59]. Another important pro-angiogenic member of this family is CXCL12 that is likely to be derived from specialized stromal cells and tumor cells [59]. Angiostatic chemokines (ELR^−^) include platelet factor 4 (CXCL4/PF) -4, CXCL4L1/PF-4var, CXCL9/Mig, CXCL10/IP-10, CXCL11/I-TAC and CXCL14/BRAK [60]. Main receptor for angiostatic CXC ELR^−^ chemokines is CXCR3. It can bind to CXCL4/PF-4, CXCL9/Mig, CXCL10/IP-10 and CXCL11/I-TAC [61]. CXCL4/PF-4, a second major platelet chemokine, is the first member of this family described to have angiostaticbehaviour. It is stored alongside with other secreteable platelet proteins including pro-angiogenic chemokines [62]. Binding of pro-angiogenic factors (VEGF and bFGF) to their respective receptors on surface of ECs are inhibited by CXCL4/PF-4 and also halts cell cycle progression [62]. CXCL9 and CXCL10 are other members of this group which have been shown to inhibit various stages of angiogenesis invitro and in vivo assays [63]. CXCL10 inhibits CXCL8- and FGF-2-mediated angiogenesis [55].

#### 3.3.1. Tumor Necrosis Factor α (TNF-α)

Like TGF-β, controversial reports exist on the role of TNF-α in angiogenesis [64]. Studies have shown that TNF-α inhibits angiogenic sprouting at higher concentrations, while at lower concentration stimulates angiogenesis cascade by inducing “tip cell” phenotype in ECs through an NF-κB dependent mechanism [65]. TNF-α delays the VEGF-driven angiogenic response by blocking signalling through VEGFR2 besides also up-regulating the expression of granulocyte-macrophage-colony stimulating factor (GM-CSF), interleukin-1 (IL-1), platelet-derived growth factor B (PDGFB) and vascular endothelial cell growth factor receptor-2 (VEGFR2), all at the same time [66]. Thus, the temporary expression of TNF in angiogenesis is critical: angiogenesis is delayed by initially blocking VEGFR2 signalling, while inducing a tip cell phenotype through NF-κB dependent mechanism, it concurrently prompt the endothelial cells (ECs) for sprouting once initial inflammatory phase has passed [53]. TNF-α and LPS exposure led to the up-regulation of VEGF and SIRT1 with subsequent up-regulation of MMP-2 and MMP-9 production to promote angiogenesis via pathways involving PI3K, p38, ERK, JNK and NF-κB was understood from another study that was using human dental papilla cells (HDPCs) [67]. Thalidomide is an antiangiogenic agent and targets TNF-α in AML myeloid metastasis [68].

#### 3.3.2. Interferon Alpha (IFN-α)

IFN-α is a cytokine which has been shown to exhibit broad spectrum pharmacological activities including angiogenesis arresting. It has been shown to inhibit EC motilityand survivalby blocking activity of angiogenic molecules including bFGF, IL-8, and MMP-9 [69]. In vivo xenograft studies have shown that it arrest tumor growth via different mechanisms in different animal models. In subcutaneous xenograft models anti-angiogenic effects of IFN-α appear to be associated with increased hypoxia and ischemic necrosis, while in transgenic mouse models, IFN-α has been shown to simultaneously target both blood vessels and tumor cell proliferation, leading to regression of tumors without necrosis [70].

#### 3.3.3. Monocyte Chemotactic Protein-1 (MCP-1)

MCP-1, a key CC chemokine has been shown to have angiogenic activities in a variety of in vitro and in vivo assays. It has been shown to play a central role in inflammation and angiogenesis and controls trafficking and activation of monocytes/macrophages through its receptor CCR2. Studies have revealed that MCP-1 can directly act on ECs to induce angiogenesis. In vivo studies using rabbit and porcine models have resulted in increased monocyte/macrophage recruitment, collateral vessel formation, and blood flow to ischemic tissue in hind limb models of ischemia through exogenous administration of MCP-1 [71].

#### 3.3.4. Hepatocyte Growth Factor (HGF) and C-Met

HGF and its receptor c-Met are involved in a communicative interplay between HGF-producing mesenchymal cells and c-Met-expressing target cells [72]. HGF is an angiogenic growth factor and its mitogenic effect in vitroin endothelial cellsis even more than that elicited by VEGF and bFGF [73]. HGF and c-Met receptor are essential to induce regeneration of endothelial cells and neovascularization during myocardial infarction [74]. The combination of HGF and VEGF has an additive effect on migration of endothelial cells and enhanced neovascularization in vivo [74]. Maturation of blood and collateral vessels promote angiopoietin/Tie2 ligand receptor system [75]. Notably, angiopoietin promotes HGF and induces the recruitment of smooth muscle cells, enhancing the stabilization of angiogenesis [72]. Previous studies have proven that HGF induced c-Met activation plays a fundamental role in angiogenesis and tumour progression in colorectal cancer by avoiding anti-angiogenic therapy and maintaining the glucose uptake and utilization by inducing GLUT1 expression [76]. In addition, inhibition of HGF and c-Met signaling pathway has resulted in tumor reduction and progression in pancreatic cancer which paves the way for effective therapeutic approach [77].

### 3.4. Membrane Protein and Adhesion Proteins

#### 3.4.1. Ephrins (Eph)

Ephrins are ligands of ephrin receptors that are involved in angiogenesis via contact–dependent cell-cell communication [78]. Eph receptors are considered largest receptor families in tyrosine kinase receptors that are required for blood vessel maturation and vascular remodelling during embryonic development [79]. Eph receptors can be divided into two groups, namely EphA and EphB receptors, with EphA2/ephrinA1 and EphB4/ephrinB2 playing important roles in vasculogenesis and angiogenesis [80]. Both receptors are required for vascular development during embryonic stage [78]. Reciprocal function of both EphB4/ephrinB2 promotes proper spatial position and vessel assembly during angiogenesis. On the other hand, EphA2 and ephrinA1 are found to correspond to regions of blood vessel formation, and may play a role in enhancing angiogenesis [80]. Nevertheless, ephrin protein expression may be different in tumor vessels due to poor distinction and organization of arterial and venous nature in tumors [78]. Regorafenib is an antiangiogenic agent and targets EGFR in metastatic colon cancer [81].

#### 3.4.2. Semaphorins

Semaphorins and its receptors are a large family of secretory and membrane bound proteins that regulate various biological processes including angiogenesis and cancer progression [82]. Semaphorins are classified into eight classes with two major receptor families, neuropilins and plexins [82]. It was reported that semaphorins class three inhibited VEGF by competing with VEGF to bind to neuropilins. Another study reported that the transcription repressor zinc finger E-box binding homeobox-box (ZEB)-1 was highly expressed in lung cancer after inhibiting semaphorins class three [83]. Re-expression of semaphorins class three in the tumors results in reduced tumor hypoxia and in vessel normalization [82].

#### 3.4.3. Integrins

Integrins are a class of heterodimer adhesion molecules comprising of isoforms of α and β subunits. At least eight heterodimeric integrins (α1β1, α2β1, α3β1, α6β1, α6β4, α5β1, αvβ3, αvβ5) have been identified on ECs; each of these heterodimer recognizes specific ligands in extracellular matrix (ECM). These transmembrane glycoproteins are highly expressed in newly-formed blood vessels and known to play important role during cell-cell and cell-ECM interactions. Integrins are involved in regulation of many physiologic processes, such as inflammation, immunity, hemostasis, wound healing, tissue differentiation, regulation of cell growth, and angiogenesis. Abnormalities in integrin signalling have been shown to promote many diseases including autoimmune diseases, thrombotic disorders, and cancer. Many lines of evidences have shown that these integrin heterodimers act via different mechanisms to promote angiogenesis. bFGF is required for αvβ3-mediated angiogenesis while integrin αvβ5 requires VEGF-to induce its pro-angiogenic effects [84]. Apart from promoting angiogenesis, these molecules are also known to suppress apoptosis in ECs [85]. When α5β1 and αvβ3 inhibit caspase-8 activation, the expression of Bcl-2, an anti-apoptotic protein, is upregulated, which suppresses protein kinase A (PKA) activity, which is required for caspase-8 activation causing cells to be deprived of integrin-mediated adhesion to the ECM, leading to the cells to rapidly undergo apoptosis. [86]. Other integrins, such as αvβ3 and α5β1, promote cell survival by suppressing p53 activity and activation of nuclear factor κB and Shc pathways [87].

#### 3.4.4. Vascular Endothelial (VE)-Cadherin

Also known as cadherin-5, this is an important member of the cadherin superfamily of transmembrane molecules, which play a key role in endothelium integrity, control of vascular permeability and in a variety of cell-cell interactions. Role of VE-cadherins in tumor-associated angiogenesis has been highlighted by numerous studies. Earlier studies have shown that blocking function of this cadherin by antibodies resulted in blockade of neovascularization at various primitive stages in embryos of mice indicating that VE-cadherin is required for developmental angiogenesis [88]. It was reported that VE-cadherin disassembly and cell contractility endothelium are necessary for barrier disruption induced by tumor cells [89].

#### 3.4.5. Platelet Endothelial Cell Adhesion Molecule-1 (PECAM-1 or CD31)

PECAM-1, also known ascluster of differentiation 31 (CD31) is a protein from immunoglobulin (Ig) superfamily and is expressed in wide range of cells within the vascular compartment including ECs, platelets, macrophages, Kupffer cells, granulocytes, T/NK cells, lymphocytes, megakaryocytes, neutrophils and osteoclasts [90,91]. In ECs it is present in abundance at intracellular junctions of adjacent cells where it serves the purpose of adhesion thus keeping cells together [92]. This cell-cell interaction has been shown to be necessary for in vitro organization of ECs into tubular networks.Administration of antibody against PECAM-1, has been shown to block in vitro differentiation rat capillary ECs into a tube-like network. Moreover, it also blocked bFGF-induced rat corneal neovascularization thus preventing angiogenesis [52].

### 3.5. Matrix Degrading Enzyme

#### Matrix Metalloproteinases (MMPs)

MMPs, also known as matrixins, are a family of enzymes which have capacity to degrade various components of extracellular matrix (ECM). However, studies have revealed that MMPs’ control multiple phases of angiogenic cascade including release of ECM-sequestered pro-angiogenic factors, release of ECM bound growth factors and receptors, including integrins and adhesion receptors, and release of endogenous inhibitors of angiogenesis [93]. To thisdate, 20 members of this family have been identified. Out of these, MMP-2 and -9 which are synthesized and secreted in large amounts by tumor cells in a paracrine and/or autocrine manner, have been extensively studied. These enzymes are known to play a critical role in the “angiogenic switch”, increasing release of VEGF, thus shifting balance towards increased tumor angiogenesis. A strong positive correlation between MMP-2, -9 and VEGF exists in majority of solid tumors leading to remodelling of ECM, increased EC proliferation, migration and vessel sprouting [94]. Studies revealed that MMP-9 is required for shift of angiogenic balance towards pro-angiogenic phase while MMP-2 contributes in tumor growth. Exogenous MMP-9 has been revealed to enhance EC growth in vitro, and is shown to increase VEGF releasefrom ECM. In addition it is also involved in recruitment of pericytes to newly formed blood vessels [93].

### 3.6. Small Mediators

#### 3.6.1. Histamine and Serotonin

Histamine and serotonin (5-hydroxytryptamine [5-HT]) are biogenic amines which have been shown to play a key role in the regulation of multiple essential processes in in vivo and cultured cells. Histamine is mostly found in mast cells and basophils, macrophages, parietal cells of stomach, cancer cells and mammalian tissues by neurons. Serotonin is widely expressed in dense granules of platelets and granules of mast cells along with histamine. Studies have shown that these amines have dual effect on angiogenesis cascade. At first exposure, these amines induce angiogenesis which is dependent on TR3/Nur77 signalling. These amines act on HUVECs and induce proliferation, migration, and tube formation in in vitro assays and reduce the expression of thrombspondin-1 (TSP-1), a potent angiogenesis inhibitor. However, these effects are transient which are followed by up-regulation of TSP-1 promoter and restoration of TSP-1 levels to normal. This trigger a negative feedback loop leading to regression of vasculature and limiting the angiogenic response induced by histamine and serotonin [95].

#### 3.6.2. Endostatin

Endostatin is a 20 kDa carboxyl-terminal proteolytic fragment of type XVIII collagen. It inhibits angiogenesis under different pathological conditions distinguished by increased angiogenesis and acts as a potent endogenous inhibitor of angiogenesis in cancer and many other experimental models. Endostatin has been reported to interfere with VEGF/VEGFR signalling and suppresses TNF-α, FGF-2 mediated angiogenesis leading to inhibition of ECs proliferation, migration/invasion, differentiation into tubes and increased apoptosis [96,97].

#### 3.6.3. Angiostatin

Angiostatin, a 38 kDa amino terminal fragment of plasminogen, is another endogenous inhibitor of angiogenesis which needs to be cleaved by various proteases to be activated. It has been shown to have both potent antiangiogenic activity and anti-proliferative activities in both endothelial and cancer cells. It acts on ECs and blocks multiple steps in angiogenic cascade including proliferation, migration and differentiation into tube-like structures inin vitromodels. It also inhibits HGF stimulated migration and proliferation of smooth muscle and ECs but has little effect on VEGF or bFGF-induced angiogenesis cascade [98]. Adding to that, it also interrupts G2/M phase of cell cycle in these cells [99]. In vivo angiostatin has been shown to strongly block neovascularisation and tumor metastasis [100]. Mechanistically it is proposed to bind with subunits of ATP synthase in ECs thereby rendering them out of ATP supply and thus inhibit proliferation. It also binds with integrin α_γ_β_3_ and block angiogenic signalling through this pathway [52].

#### 3.6.4. Thrombospondins (TSPs) 

TSPs were first identified by Jack Lawler and colleagues in 1977 in platelets treated with thrombin. Since then extensive research is being conduct on these molecules and till date five member of this family (TSP 1–5) have been identified. TSP-1 and -2 have been shown to have anti-angiogenic activity owing to their “type I repeats” which play a major role in anti-angiogenic properties of TSP-1 and -2 [101]. TSP-1, has been revealed to be synthesized and secreted by a wide variety of ECs from different sources including aortic, venous, capillary, and corneal endothelial cells as well as from fibroblasts and smooth muscle cells. Both TSP-1 and -2 directly neutralize the activity of VEGF on EC, arresting cell migration, proliferation, survival, and promote apoptosis [102]. CD36, CD47, and integrins are the channel from which TSP-1 and TSP-2 exert their direct effects [103]. Other than that, these receptors appear to associate with VEGFR2 to form a platform that receives positive and negative signals for angiogenesis [103]. Cross talk between pro- and anti-angiogenic signal transduction pathways shows by antagonizing survival pathways while also activating apoptotic pathways, it may enable TSP-1 and TSP-2 to inhibit angiogenesis [104].

#### 3.6.5. Galectins (Gals)

Galectins are from the lectin family. They show high affinity for β-galactosides [105]. Gals have been found in almost every cell and they play a fundamental role in cell signaling, proliferation, migration, apoptosis, and mRNA splicing. Galectins are classified intothree groups based on their structure, the prototype galectins, chimeric galectins, and the tandem repeat galectins. Among all the galectins, plenty of focus has been placed onto Gal-1, -3, -8, -9 [106,107,108]. Galectin-targeted angiostatic therapy can be aimed at scavenging the secreted angiostimulatory galectins and thus block their coupling with oligosaccharide chains in endothelial cells [109,110]. For instance, gal-1 was found to be involved in VEGFR2 signalling in tumors which promoted the secretion of the growth factor leading to angiogenesis [106,111]. Gal-3 induce the release of pro-inflammatory cytokine such as IL-6, G-CSF, GM-CSF, and sICAM-1 from endothelial cells, increasing endothelial cell surface adhesion molecules leading to the promotion of metastasis that improved cell tube formation which portrayed angiogenesis [107,108]. There is limited information regarding gal-8 and gal-9, however it is understood that gal-8 stimulated tube formation and migration of EC while gal-9 was involved in sprouting [111]. There is much research still need to be done regarding gal-8 and -9 in cancer.

### 3.7. MicroRNA (miRNAs)

Mounting evidence indicates that miRNAs play a key role in diverse biological processes but in cancer particularly, miRNAs play a key role in tumorigenesis, angiogenesis and have oncogenic or tumor suppressor roles [112,113]. miRNAs can be classified as pro-angiogenic or anti-angiogenic, among them most are predominantly pro-angiogenic. miRNA-155 was found to be frequently overexpressed in different types of cancer and the ectopic expression of miRNA-155 upregulated angiogenesis in the cancer [114]. miRNA-296 is involved in regulation of PDGFR, EGFR and VEGFR signalling pathways [115]. In vivo studies demonstrated that down-regulation of the miRNA-126 stimulates Sprouty Related EVH domain containing protein 1 (SPRED-1) and phosphoinositol-3kinase regulatory subunit 2 (PIK3R2/p85-beta), which are both negative regulators of the VEGF/VEGFR signalling pathway [116,117]. miRNA-7 was recently identified as an anti-angiogenic miRNA and targets the EGFR and PI3K signalling pathways. It inhibits angiogenesis and also tumor cell proliferation [118].

## 4. Future Perspectives

Pre-clinical and clinical data shows that anti-angiogenic strategy holds a promise to treat some cancer types especially solid tumors. Although this technique still needs to optimize and study more to make sure there is no side effect and risky using it. It is very important to understand how and why different target genes are activated according to cell type and angiogenic tumor. Anti-angiogenic therapies have been used as anticancer therapy and there are a number of potential anti-angiogenic compounds that have been shown to suppress the growth of tumor and metastasis. Theses inhibitors function by blocking the activity of growth factors either by binding to the ligands or by preventing the interaction with the VEGF receptors and their ligands (Table 1). Therapies targeting not only tumor cells or EC cells but also TAMs are highly demanded keeping in view the present complex scenario of cancer progression. This idea is based on broad spectrum targeting of multiple targets in cancer thus arresting multiple phases of tumor progression. Innovations in experimental treatment of cancers are thanks to the advances in understanding the molecular regulation of angiogenesis. This has paved the way for future improvements in the field of cancer treatment study and will likely continue to offer vast avenues for discovery in other disease processes as well.

## 5. Conclusions

Currently, with a small number of identified targets in different cell type and tissue, it is difficult to predict a particular gene therapy to specific cell or tumor. Therefore, understanding the role of these target genes in normal cells and tumors will allow developing drugs that specific for some target genes. However, no definitive tumor biomarker has been identified yet. Therefore, angiogenesis therapy would be of considerable therapeutic potential in treatment of cancer as well as it can help to stop tumor growth and cease tumor metastasis. Such angiogenesis therapy well gives an accurate prognostic indicator that helps to conclude which patients may need aggressive adjuvant therapy. Anti-growth factors will probably help to treat angiogenesis tumors or disturb growing these tumors and prevent metastasis.

## Figures and Tables

**Table 1:** List of anti-angiogenic agents.

No	Antiangiogenic Agent	Target/Targets	Cancer Type	References
1	ABP 215	VEGF	Metastatic non-squamous NSCLC	[32,119]
2	Apatinib	VEGF and VEGFR-2	Advanced or metastatic gastric cancer, advanced non-squamous non small cell lung cancer, colorectal cancer, metastatic esophageal cancer, advanced pancreatic cancer, advanced and metastatic breast cancer, metastatic renal cell carcinoma, and thyroid cancer Platinum-resistant or refractory ovarian cancer	[32,33]
3	Axitinib	VEGF-1, 2, and 3	Renal cell carcinoma	[120,121]
4	Bevacizumab	VEGF	Metastatic colorectal cancer, non-squamous, non-small cell lung cancer and metastatic breast cancer	[34,122,123]
5	Bortezomib	NF-κB and VEGF	Multiple myeloma (MM) and mantle cell lymphoma	[124,125]
6	Cabozantinib	RET, MET, VEGFR-(1,2,and 3), KIT, TRKB, FMS-like tyrosine kinase-3(FLT3), AXL ROS1, TYRO3, and TIE-2	Progressive, metastatic medullary thyroid cancer, Advanced renal cell carcinoma	[32,126]
7	Cediranib	VEGFR1, VEGFR2 PDGFR-β, and VEGFR-3	Prostate, pancreatic, colon, breast, neck, renal cancers, ovarian and AML	[45,127]
8	Glesatinib	c-MET and AXL	Non-small cell lung cancer and head and neck squamous cell carcinoma	[128]
9	Emibetuzumab	FGF and HGF	Gastric cancer	[39]
10	Everolimus	mTOR	HER2-HR+ breast cancer Advanced renal cell carcinoma Pancreatic GI-NETNET Lung NET Subependymal giant cell astrocytoma	[32,129]
11	Lenalidomide	VEGF and Interleukin-6	Multiple myeloma, primary myelofibrosis, and myeloid metastasis	[130,131]
12	Imatinib	VEGF, PDGF PDGF, SCF, c-kit, and BCR-ABL	Chronic myeloid leukemia (CML), gastrointestinal stromal tumor (GIST), and Philadelphia chromosome-positive (Ph+) acute lymphoblastic leukemia	[123]
13	Lenvatinib	VEGFR-(1, 2, and 3), FGFR-(1, 2, 3, and 4), PDGFR-alpha, KIT, and RET	Differentiated thyroid cancer renal cell cancer	[32,132]
14	Lucitanib	VEGFR-(1, 2, 3) and FGFR-(1, 2)	Metastatic breast cancer	[32,133]
15	Olaparib	PARP and VEGFR	ovarian cancer	[134]
16	Pazopanib	VEGFR-1, -2, -3, PDGFR-alpha, PDGFR-beta, FGFR-1, -3, KIT, LTK, Lck, c-Fms	Advanced renal cell carcinoma Advanced soft tissue sarcoma	[32,135]
17	Ponatinib	ABL, VEGFR, PDGFR, FGFR, EPH receptors, SRC, KIT, RET, TIE2, FLT3	Chronic myeloid leukemia Acute lymphoblastic leukemia	[32,136]
18	Ramucirumab	VEGFR-2	Metastatic colorectal Metastatic NSCLC Advanced or metastatic gastric or gastroesophageal junction adenocarcinoma	[32,137]
19	Regorafenib	RET, VEGFR-1, -2, -3, KIT, PDGFR-alpha and beta, FGFR-1, -2, TIE2, DDR2, TrkA, Eph2A, RAF-1, BRAF and BRAFV600E, SAPK2, PTK5, Abl	Metastatic colorectal cancer locally advanced, unresectable, or metastatic GIST	[32,81]
20	Sorafenib	VEGFR-2 and -3, PDGFR-b, FLT3, and c-Kit VEGFR-1, -2, -3, PDGFR-beta, KIT, FLT3, RET, RET/PTC	Unresectable Hepatocellular carcinoma Advanced renal cell carcinoma Locally recurrent or metastatic, progressive, and differentiated thyroid carcinoma	[46]
21	Sunitinib	VEGFR-1, -2, -3, PDGFR-alpha and beta, KIT, FLT3, CSF-1R, RET	Advanced and metastatic renal cell carcinoma	[138]
22	SU5416 (Semaxinib)	VEGFR-(1 and 2), c-kit, and FLT3	Advanced acute myeloid leukemia (AML) and myelodysplastic syndromes	[139,140]
23	Temsirolimus	mTOR	Advanced renal cell carcinoma	[141]
24	Thalidomide	TNF-α synthesis	AML myeloid metastasis	[68]
25	Vandetanib	VEGFR, EGFR, RET, BRK, TIE2, EPH receptor, SRC kinase	Symptomatic or progressive medullary thyroid cancer	[142]
26	Vatalanib	VEGFR and PDGFR tyrosine kinases	Breast, colorectal carcinoma, liver metastasis, AML, PMF, blast phase of chronic myelogenous leukemia, and myelodysplastic syndromes (MDS) 7374	[50,143]
27	Ziv-aflibercept	VEGF-(A and B) and PIGF	Metastatic non-squamous non-small cell lung cancer	[144]

EGF: vascular endothelial growth factor; NSCLC: non small cell lung cancer; PDGFR: platelet-derived growth factors receptor; VEGFR: vascular endothelial growth factor receptor; NF: necrosis factor; RET: rearrange during transfection; MET: mesenchymal-epithelial transition factor; KIT: cellular homolog of the transforming gene of a feline retrovirus; TRKB: Tropomyosin receptor kinase B; FLT3: FMS-like tyrosine kinase-3; AXL: anexelekto; ROS: Proto-oncogene tyrosine-protein; TYRO: tyrosine kinase-binding protein; TIE: Tyrosine Kinase With Immunoglobulin And Epidermal Growth Factor Homology Domains; FGF: fibroblast growth factor; HGF: hepatocyte growth factor; mTOR: mammalian target of rapamycin; SCF: Stem cell factor; BCR-ABL: breakpoint cluster region protein-Abelson murine leukemia viral oncogene homolog 1; HER-2-HR+: human epidermal growth factor receptor 2-positive breast cancer; GI-NET: Gastrointestinal neuroendocrine tumors; LTK: Leukocyte Receptor Tyrosine Kinase; Lck: lymphocyte specific protein tyrosine kinase; c-Fms: macrophage colony-stimulating factor receptor (M-CSFR); EPH: Ephrin; SRC: Steroid Receptor Coactivator; DDR2: discoidin domain-containing receptor 2 precursor; TrkA: tropomyosin receptor kinase A; Eph2A: ephrin type-A receptor 2; RAF-1: proto-oncogene serine/threonine-protein kinase; BRAF: serine/threonine-protein kinase B-Raf; SAPK2: Serine/threonine-protein kinase 2; PTK5: protein tyrosine kinase 5; PTC: phenylthiocarbamide; CSF-1R: colony stimulating factor 1 receptor; PMF: primary myelofibrosis.

## References

[1] Folkman J., Merler E., Abernathy C., Williams G. (1971). Isolation of a tumor factor responsible for angiogenesis. J. Exp. Med..

[2] Davis G.E., Senger D.R. (2005). Endothelial extracellular matrix biosynthesis, remodeling, and functions during vascular morphogenesis and neovessel stabilization. Circ. Res..

[3] Baldewijns M., Thijssen V., Van den Eynden G., Van Laere S., Bluekens A., Roskams T., Van Poppel H., De Bruine A., Griffioen A., Vermeulen P. (2007). High-grade clear cell renal cell carcinoma has a higher angiogenic activity than low-grade renal cell carcinoma based on histomorphological quantification and qRT–PCR mRNA expression profile. Br. J. Cancer.

[4] Morikawa S., Baluk P., Kaidoh T., Haskell A., Jain R.K., McDonald D.M. (2002). Abnormalities in pericytes on blood vessels and endothelial sprouts in tumors. Am. J. Pathol..

[5] Ruoslahti E. (2002). Specialization of tumour vasculature. Nat. Rev. Cancer.

[6] Monahan-Earley R., Dvorak A., Aird W. (2013). Evolutionary origins of the blood vascular system and endothelium. J. Thromb. Haemost..

[7] Koh G.Y., Joo H.J., Choi D.K., Park J.-S., Cho S.W. (2015). Method for Forming Endothelial Cells. U.S. Patent.

[8] Williams P.A. (2016). Manipulating Endothelial Progenitor Cell Homing with Sphingosine-1-Phosphate for Terapeutic Angiogenesis.

[9] Yehya A.H., Asif M., Tan Y.J., Sasidharan S., Majid A.M.A., Oon C.E. (2017). Broad spectrum targeting of tumor vasculature by medicinal plants: An updated review. J. Herb. Med..

[10] Carmeliet P., Jain R.K. (2000). Angiogenesis in cancer and other diseases. Nature.

[11] Oon C.E., Bridges E., Sheldon H., Sainson R.C.A., Jubb A., Turley H., Leek R., Buffa F., Harris A.L., Li J.L. (2017). Role of Delta-like 4 in Jagged1-induced tumour angiogenesis and tumour growth. Oncotarget.

[12] Carmeliet P., Jain R.K. (2011). Principles and mechanisms of vessel normalization for cancer and other angiogenic diseases. Nat. Rev. Drug Discov..

[13] Eelen G., de Zeeuw P., Simons M., Carmeliet P. (2015). Endothelial cell metabolism in normal and diseased vasculature. Circ. Res..

[14] Klemm F., Joyce J.A. (2015). Microenvironmental regulation of therapeutic response in cancer. Trends Cell Biol..

[15] Wang Z., Dabrosin C., Yin X., Fuster M.M., Arreola A., Rathmell W.K., Generali D., Nagaraju G.P., El-Rayes B., Ribatti D. (2015). Broad targeting of angiogenesis for cancer prevention and therapy. Semin. Cancer Biol..

[16] Sun W. (2012). Angiogenesis in metastatic colorectal cancer and the benefits of targeted therapy. J. Hematol. Oncol..

[17] Siemann D.W. (2011). The unique characteristics of tumor vasculature and preclinical evidence for its selective disruption by tumor-vascular disrupting agents. Cancer Treat. Rev..

[18] Balkwill F.R., Capasso M., Hagemann T. (2012). The tumor microenvironment at a glance. J. Cell Sci..

[19] Davies L.C., Jenkins S.J., Allen J.E., Taylor P.R. (2013). Tissue-resident macrophages. Nat. Immunol..

[20] Sica A., Mantovani A. (2012). Macrophage plasticity and polarization: In vivo veritas. J. Clin. Investig..

[21] Jetten N., Verbruggen S., Gijbels M.J., Post M.J., De Winther M.P., Donners M.M. (2014). Anti-inflammatory M2, but not pro-inflammatory M1 macrophages promote angiogenesis in vivo. Angiogenesis.

[22] Zhong W.-Q., Chen G., Zhang W., Xiong X.-P., Zhao Y., Liu B., Zhao Y.-F. (2015). M2-polarized macrophages in keratocystic odontogenic tumor: Relation to tumor angiogenesis. Sci. Rep..

[23] Nielsen S.R., Schmid M.C. (2017). Macrophages as key drivers of cancer progression and metastasis. Mediat. Inflamm..

[24] Yoo S.Y., Kwon S.M. (2013). Angiogenesis and its therapeutic opportunities. Mediat. Inflamm..

[25] Olsson A.-K., Dimberg A., Kreuger J., Claesson-Welsh L. (2006). VEGF receptor signalling? In control of vascular function. Nat. Rev. Mol. Cell Biol..

[26] Hicklin D.J., Ellis L.M. (2005). Role of the vascular endothelial growth factor pathway in tumor growth and angiogenesis. J. Clin. Oncol..

[27] Nakayama M., Nakayama A., van Lessen M., Yamamoto H., Hoffmann S., Drexler H.C., Itoh N., Hirose T., Breier G., Vestweber D. (2013). Spatial regulation of VEGF receptor endocytosis in angiogenesis. Nat. Cell Biol..

[28] Terme M., Pernot S., Marcheteau E., Sandoval F., Benhamouda N., Colussi O., Dubreuil O., Carpentier A.F., Tartour E., Taieb J. (2013). VEGFA-VEGFR pathway blockade inhibits tumor-induced regulatory T-cell proliferation in colorectal cancer. Cancer Res..

[29] Kubatka P., Kapinová A., Kello M., Kruzliak P., Kajo K., Výbohová D., Mahmood S., Murin R., Viera T., Mojžiš J. (2016). Fruit peel polyphenols demonstrate substantial anti-tumour effects in the model of breast cancer. Eur. J. Nutr..

[30] Martins S.F., Garcia E.A., Luz M.A.M., Pardal F., Rodrigues M., Longatto Filho A. (2013). Clinicopathological correlation and prognostic significance of VEGF-A, VEGF-C, VEGFR-2 and VEGFR-3 expression in colorectal cancer. Cancer Genom. Proteom..

[31] Matsumoto M., Roufail S., Inder R., Caesar C., Karnezis T., Shayan R., Farnsworth R.H., Sato T., Achen M.G., Mann G.B. (2013). Signaling for lymphangiogenesis via VEGFR-3 is required for the early events of metastasis. Clin. Exp. Metastasis.

[32] Siemann D., Chaplin D., Horsman M. (2017). Realizing the potential of vascular targeted therapy: The rationale for combining vascular disrupting agents and anti-angiogenic agents to treat cancer. Cancer Investig..

[33] Li J., Qin S., Xu J., Xiong J., Wu C., Bai Y., Liu W., Tong J., Liu Y., Xu R. (2016). Randomized, double-blind, placebo-controlled phase III trial of apatinib in patients with chemotherapy-refractory advanced or metastatic adenocarcinoma of the stomach or gastroesophageal junction. J. Clin. Oncol..

[34] Miller K., Wang M., Gralow J., Dickler M., Cobleigh M., Perez E.A., Shenkier T., Cella D., Davidson N.E. (2007). Paclitaxel plus bevacizumab versus paclitaxel alone for metastatic breast cancer. N. Engl. J. Med..

[35] Baird A., Esch F., Mormede P., Ueno N., Ling N., Bohlen P., Ying S., Wehrenberg B., Guillemin R. (1986). Molecular characterization of fibroblast growth factor: Distribution and biological activities in various tissues. Recent Prog. Horm. Res..

[36] Cross M.J., Claesson-Welsh L. (2001). FGF and VEGF function in angiogenesis: Signalling pathways, biological responses and therapeutic inhibition. Trends Pharmacol. Sci..

[37] Greaves N.S., Ashcroft K.J., Baguneid M., Bayat A. (2013). Current understanding of molecular and cellular mechanisms in fibroplasia and angiogenesis during acute wound healing. J. Dermatol. Sci..

[38] Presta M., Chiodelli P., Giacomini A., Rusnati M., Ronca R. (2017). Fibroblast growth factors (FGFs) in cancer: FGF traps as a new therapeutic approach. Pharmacol. Ther..

[39] Bendell J., Fuchs C., Voss M., Bauer T.M., Choueiri T.K., Drilon A., Thorn K., Wijayawardana S., Moser B., Uruñuela A. Abstract CT090: A Phase 1b/2 Study of Ramucirumab in Combination with Emibetuzumab in Patients with Advanced Solid Tumors. Proceedings of the AACR Annual Meeting.

[40] Jitariu A.A., Cimpean A.M., Kundnani N.R., Raica M. (2015). State of the art paper Platelet-derived growth factors induced lymphangiogenesis: Evidence, unanswered questions and upcoming challenges. Arch. Med. Sci..

[41] Farooqi A.A., Siddik Z.H. (2015). Platelet-derived growth factor (PDGF) signalling in cancer: Rapidly emergingsignalling landscape. Cell Biochem. Funct..

[42] Hill R.A., Patel K.D., Medved J., Reiss A.M., Nishiyama A. (2013). NG2 cells in white matter but not gray matter proliferate in response to PDGF. J. Neurosci..

[43] Jurek A., Amagasaki K., Gembarska A., Heldin C.-H., Lennartsson J. (2009). Negative and positive regulation of MAPK phosphatase 3 controls platelet-derived growth factor-induced Erk activation. J. Biol. Chem..

[44] Heldin C.-H. (2013). Targeting the PDGF signaling pathway in tumor treatment. Cell Commun. Signal..

[45] Drevs J., Siegert P., Medinger M., Mross K., Strecker R., Zirrgiebel U., Harder J., Blum H., Robertson J., Jürgensmeier J.M. (2007). Phase I clinical study of AZD2171, an oral vascular endothelial growth factor signaling inhibitor, in patients with advanced solid tumors. J. Clin. Oncol..

[46] Llovet J.M., Ricci S., Mazzaferro V., Hilgard P., Gane E., Blanc J.-F., de Oliveira A.C., Santoro A., Raoul J.-L., Forner A. (2008). Sorafenib in advanced hepatocellular carcinoma. N. Engl. J. Med..

[47] Hong M.-C., Long C.-Y., Tian Y.-F., Wu M.-P. (2013). Her-2/neu overexpression is associated with thrombospondin-1-related angiogenesis and thrombospondin-1-unrelated lymphangiogenesis in breast cancer. Gynecol. Minim. Invasive Ther..

[48] Yewale C., Baradia D., Vhora I., Patil S., Misra A. (2013). Epidermal growth factor receptor targeting in cancer: A review of trends and strategies. Biomaterials.

[49] Yan L., Wu W., Wang Z., Li C., Lu X., Duan H., Zhou J., Wang X., Wan P., Song Y. (2013). Comparative study of the effects of recombinant human epidermal growth factor and basic fibroblast growth factor on corneal epithelial wound healing and neovascularization in vivo and in vitro. Ophthalmic Res..

[50] Mross K., Drevs J., Müller M., Medinger M., Marmé D., Hennig J., Morgan B., Lebwohl D., Masson E., Ho Y.-Y. (2005). Phase I clinical and pharmacokinetic study of PTK/ZK, a multiple VEGF receptor inhibitor, in patients with liver metastases from solid tumours. Eur. J. Cancer.

[51] Geng L., Chaudhuri A., Talmon G., Wisecarver J.L., Wang J. (2013). TGF-beta suppresses VEGFA-mediated angiogenesis in colon cancer metastasis. PLoS ONE.

[52] Distler J.H., Hirth A., Kurowska-Stolarska M., Gay R.E., Gay S., Distler O. (2003). Angiogenic and angiostatic factors in the molecular control of angiogenesis. Q. J. Nucl. Med..

[53] Ucuzian A.A., Gassman A.A., East A.T., Greisler H.P. (2010). Molecular mediators of angiogenesis. J. Burn Care Res..

[54] Saenz S.A., Taylor B.C., Artis D. (2008). Welcome to the neighborhood: Epithelial cell-derived cytokines license innate and adaptive immune responses at mucosal sites. Immunol. Rev..

[55] Mehrad B., Keane M.P., Strieter R.M. (2007). Chemokines as mediators of angiogenesis. Thromb. Haemost..

[56] Sahin H., Borkham-Kamphorst E., Kuppe C., Zaldivar M.M., Grouls C., Al-samman M., Nellen A., Schmitz P., Heinrichs D., Berres M.L. (2012). Chemokine Cxcl9 attenuates liver fibrosis-associated angiogenesis in mice. Hepatology.

[57] Passaro C., Borriello F., Vastolo V., Di Somma S., Scamardella E., Gigantino V., Franco R., Marone G., Portella G. (2016). The oncolytic virus dl922-947 reduces IL-8/CXCL8 and MCP-1/CCL2 expression and impairs angiogenesis and macrophage infiltration in anaplastic thyroid carcinoma. Oncotarget.

[58] Kroeze K.L., Boink M.A., Sampat-Sardjoepersad S.C., Waaijman T., Scheper R.J., Gibbs S. (2012). Autocrine regulation of re-epithelialization after wounding by chemokine receptors CCR1, CCR10, CXCR1, CXCR2, and CXCR3. J. Investig. Dermatol..

[59] Gao Y., Guan Z., Chen J., Xie H., Yang Z., Fan J., Wang X., Li L. (2015). CXCL5/CXCR2 axis promotes bladder cancer cell migration and invasion by activating PI3K/AKT-induced upregulation of MMP2/MMP9. Int. J. Oncol..

[60] Zhu Q., Han X., Peng J., Qin H., Wang Y. (2012). The role of CXC chemokines and their receptors in the progression and treatment of tumors. J. Mol. Histol..

[61] Datta D., Flaxenburg J.A., Laxmanan S., Geehan C., Grimm M., Waaga-Gasser A.M., Briscoe D.M., Pal S. (2006). Ras-induced modulation of CXCL10 and its receptor splice variant CXCR3-B in MDA-MB-435 and MCF-7 cells: Relevance for the development of human breast cancer. Cancer Res..

[62] Kiefer F., Siekmann A.F. (2011). The role of chemokines and their receptors in angiogenesis. Cell. Mol. Life Sci..

[63] Keeley E.C., Mehrad B., Strieter R.M. (2011). Chemokines as mediators of tumor angiogenesis and neovascularization. Exp. Cell Res..

[64] Meng X.-M., Chung A.C., Lan H.Y. (2013). Role of the TGF-β/BMP-7/Smad pathways in renal diseases. Clin. Sci..

[65] Rao N., Lee Y.F., Ge R. (2015). Novel endogenous angiogenesis inhibitors and their therapeutic potential. Acta Pharmacol. Sin..

[66] Barrientos S., Stojadinovic O., Golinko M.S., Brem H., Tomic-Canic M. (2008). Growth factors and cytokines in wound healing. Wound Repair Regen..

[67] Shin M.R., Kang S.K., Kim Y.S., Lee S.Y., Hong S.C., Kim E.C. (2015). TNF-α and LPS activate angiogenesis via VEGF and SIRT1 signalling in human dental pulp cells. Int. Endod. J..

[68] Thomas D.A., Giles F.J., Albitar M., Cortes J.E., Verstovsek S., Faderl S., O’brien S.M., Garcia-Manero G., Keating M.J., Pierce S. (2006). Thalidomide therapy for myelofibrosis with myeloid metaplasia. Cancer.

[69] Von Marschall Z., Scholz A., Cramer T., Schäfer G., Schirner M., Öberg K., Wiedenmann B., Höcker M., Rosewicz S. (2003). Effects of interferon alpha on vascular endothelial growth factor gene transcription and tumor angiogenesis. J. Nat. Cancer Inst..

[70] Indraccolo S. (2010). Interferon-α as angiogenesis inhibitor: Learning from tumor models. Autoimmunity.

[71] Hong K.H., Ryu J., Han K.H. (2005). Monocyte chemoattractant protein-1–induced angiogenesis is mediated by vascular endothelial growth factor-A. Blood.

[72] Skibinski G., Skibinska A., James K. (2001). The role of hepatocyte growth factor and its receptor c-met in interactions between lymphocytes and stromal cells in secondary human lymphoid organs. Immunology.

[73] Ding S., Merkulova-Rainon T., Han Z.C., Tobelem G. (2003). HGF receptor up-regulation contributes to the angiogenic phenotype of human endothelial cells and promotes angiogenesis in vitro. Blood.

[74] Gallo S., Sala V., Gatti S., Crepaldi T. (2014). HGF/Met axis in heart function and cardioprotection. Biomedicines.

[75] Kobayashi H., DeBusk L.M., Babichev Y.O., Dumont D.J., Lin P.C. (2006). Hepatocyte growth factor mediates angiopoietin-induced smooth muscle cell recruitment. Blood.

[76] Mira A., Morello V., Céspedes M.V., Perera T., Comoglio P.M., Mangues R., Michieli P. (2017). Stroma-derived HGF drives metabolic adaptation of colorectal cancer to angiogenesis inhibitors. Oncotarget.

[77] Pothula S.P., Xu Z., Goldstein D., Merrett N., Pirola R.C., Wilson J.S., Apte M.V. (2017). Targeting the HGF/c-MET pathway: Stromal remodelling in pancreatic cancer. Oncotarget.

[78] Lisabeth E.M., Falivelli G., Pasquale E.B. (2013). Eph receptor signaling and ephrins. Cold Spring Harb. Perspect. Biol..

[79] Kullander K., Klein R. (2002). Mechanisms and functions of Eph and ephrin signalling. Nat. Rev. Mol. Cell Biol..

[80] Mosch B., Reissenweber B., Neuber C., Pietzsch J. (2010). Eph receptors and ephrin ligands: Important players in angiogenesis and tumor angiogenesis. J. Oncol..

[81] Mross K., Frost A., Steinbild S., Hedbom S., Büchert M., Fasol U., Unger C., Krätzschmar J., Heinig R., Boix O. (2012). A phase I dose–escalation study of regorafenib (BAY 73-4506), an inhibitor of oncogenic, angiogenic, and stromal kinases, in patients with advanced solid tumors. Clin. Cancer Res..

[82] Gu C., Giraudo E. (2013). The role of semaphorins and their receptors in vascular development and cancer. Exp. Cell Res..

[83] Clarhaut J., Gemmill R.M., Potiron V.A., Ait-Si-Ali S., Imbert J., Drabkin H.A., Roche J. (2009). ZEB-1, a repressor of the semaphorin 3F tumor suppressor gene in lung cancer cells. Neoplasia.

[84] Singh M.K., Bhattacharya D., Chaudhuri S., Acharya S., Kumar P., Santra P., Basu A.K., Chaudhuri S. (2014). T11TS inhibits glioma angiogenesis by modulation of MMPs, TIMPs, with related integrin αv and TGF-β1 expressions. Tumor Biol..

[85] Wang J.T., Liu Y., Kan X., Liu M., Lu J.G. (2014). Cilengitide, a small molecule antagonist, targeted to integrin αν inhibits proliferation and induces apoptosis of laryngeal cancer cells in vitro. Eur. Arch. Oto-Rhino-Laryngol..

[86] Notni J., Steiger K., Hoffmann F., Reich D., Kapp T.G., Rechenmacher F., Neubauer S., Kessler H., Wester H.-J. (2016). Complementary, selective PET imaging of integrin subtypes α5β1 and αvβ3 using 68Ga-aquibeprin and 68Ga-avebetrin. J. Nucl. Med..

[87] Foubert P., Varner J.A., Shimaoka M. (2012). Integrins in Tumor Angiogenesis and Lymphangiogenesis. Integrin and Cell Adhesion Molecules.

[88] Wallez Y., Vilgrain I., Huber P. (2006). Angiogenesis: The VE-Cadherin Switch. Trends Cardiovasc. Med..

[89] Aragon-Sanabria V., Pohler S.E., Eswar V.J., Bierowski M., Gomez E.W., Dong C. (2017). VE-cadherin disassembly and cell contractility in the endothelium are necessary for barrier disruption induced by tumor cells. Sci. Rep..

[90] Müller A.M., Hermanns M.I., Skrzynski C., Nesslinger M., Müller K.-M., Kirkpatrick C.J. (2002). Expression of the endothelial markers PECAM-1, vWf, and CD34 in vivo and in vitro. Exp. Mol. Pathol..

[91] Baumann C.I., Bailey A.S., Li W., Ferkowicz M.J., Yoder M.C., Fleming W.H. (2004). PECAM-1 is expressed on hematopoietic stem cells throughout ontogeny and identifies a population of erythroid progenitors. Blood.

[92] Woodfin A., Voisin M.-B., Nourshargh S. (2007). PECAM-1: A multi-functional molecule in inflammation and vascular biology. Arterioscler. Thromb. Vasc. Biol..

[93] Rundhaug J.E. (2005). Matrix metalloproteinases and angiogenesis. J. Cell. Mol. Med..

[94] Zheng H., Takahashi H., Murai Y., Cui Z., Nomoto K., Niwa H., Tsuneyama K., Takano Y. (2006). Expressions of MMP-2, MMP-9 and VEGF are closely linked to growth, invasion, metastasis and angiogenesis of gastric carcinoma. Anticancer Res..

[95] Qin L., Zhao D., Xu J., Ren X., Terwilliger E.F., Parangi S., Lawler J., Dvorak H.F., Zeng H. (2013). The vascular permeabilizing factors histamine and serotonin induce angiogenesis through TR3/Nur77 and subsequently truncate it through thrombospondin-1. Blood.

[96] Hwang H.H., Lee D.Y. (2016). Antiangiogenic actions of heparin derivatives for cancer therapy. Macromol. Res..

[97] Venkatachalam A. (2016). Effect of Mutant Endostatin and Kringle 5 Fusion Protein on Tumor Angiogenesis.

[98] Wajih N., Sane D.C. (2003). Angiostatin selectively inhibits signaling by hepatocyte growth factor in endothelial and smooth muscle cells. Blood.

[99] Hsu H.-W. (2013). Radiosensitization of Head & Neck Carcinoma Cells by Linifanib, A Receptor Tyrosine Kinase Inhibitor.

[100] Peeters C.F., de Geus L.-F., Westphal J.R., de Waal R.M., Ruiter D.J., Wobbes T., Oyen W.J., Ruers T.J. (2005). Decrease in circulating anti-angiogenic factors (angiostatin and endostatin) after surgical removal of primary colorectal carcinoma coincides with increased metabolic activity of liver metastases. Surgery.

[101] Bornstein P. (2009). Thrombospondins function as regulators of angiogenesis. J. Cell Commun. Signal..

[102] Noh Y.-H., Matsuda K., Hong Y.-K., Kunstfeld R., Riccardi L., Koch M., Oura H., Dadras S.S., Streit M., Detmar M. (2003). An N-terminal 80 kDa recombinant fragment of human thrombospondin-2 inhibits vascular endothelial growth factor induced endothelial cell migration in vitro and tumor growth and angiogenesis in vivo. J. Investig. Dermatol..

[103] Kaur S., Martin-Manso G., Pendrak M.L., Garfield S.H., Isenberg J.S., Roberts D.D. (2010). Thrombospondin-1 inhibits VEGF receptor-2 signaling by disrupting its association with CD47. J. Biol. Chem..

[104] Lawler P.R., Lawler J. (2012). Molecular basis for the regulation of angiogenesis by thrombospondin-1 and-2. Cold Spring Harb. Perspect. Med..

[105] Méndez-Huergo S.P., Blidner A.G., Rabinovich G.A. (2017). Galectins: Emerging regulatory checkpoints linking tumor immunity and angiogenesis. Curr. Opin. Immunol..

[106] Etulain J., Negrotto S., Tribulatti M.V., Croci D.O., Carabelli J., Campetella O., Rabinovich G.A., Schattner M. (2014). Control of angiogenesis by galectins involves the release of platelet-derived proangiogenic factors. PLoS ONE.

[107] Kindt N., Journe F., Ghanem G.E., Saussez S. (2017). Galectins and carcinogenesis: Their role in head and neck carcinomas and thyroid carcinomas. Int. J. Mol. Sci..

[108] Chen C., Duckworth C., Fu B., Pritchard D.M., Rhodes J., Yu L. (2014). Circulating galectins-2,-4 and-8 in cancer patients make important contributions to the increased circulation of several cytokines and chemokines that promote angiogenesis and metastasis. Br. J. Cancer.

[109] Varinska L., Kubatka P., Mojzis J., Zulli A., Gazdikova K., Zubor P., Büsselberg D., Caprnda M., Opatrilova R., Gasparova I. (2017). Angiomodulators in cancer therapy: New perspectives. Biomed. Pharmacother..

[110] Wdowiak K., Francuz T., Gallego-Colon E., Ruiz-Agamez N., Kubeczko M., Grochoła I., Wojnar J. (2018). Galectin targeted therapy in oncology: Current knowledge and perspectives. Int. J. Mol. Sci..

[111] Griffioen A.W., Thijssen V.L. (2014). Galectins in tumor angiogenesis. Ann. Transl. Med..

[112] Lages E., Ipas H., Guttin A., Nesr H., Berger F., Issartel J.-P. (2012). MicroRNAs: Molecular features and role in cancer. Front. Biosci..

[113] Cortés-Sempere M., de Cáceres I.I. (2011). microRNAs as novel epigenetic biomarkers for human cancer. Clin. Transl. Oncol..

[114] Lou W., Liu J., Gao Y., Zhong G., Chen D., Shen J., Bao C., Xu L., Pan J., Cheng J. (2017). MicroRNAs in cancer metastasis and angiogenesis. Oncotarget.

[115] Van Beijnum J.R., Giovannetti E., Poel D., Nowak-Sliwinska P., Griffioen A.W. (2017). miRNAs: Micro-managers of anticancer combination therapies. Angiogenesis.

[116] Wang L., Lee A.Y.W., Wigg J.P., Peshavariya H., Liu P., Zhang H. (2016). miR-126 regulation of angiogenesis in age-related macular degeneration in CNV mouse model. Int. J. Mol. Sci..

[117] Ji J.-S., Xu M., Song J.-J., Zhao Z.-W., Chen M.-J., Chen W.-Q., Tu J.-F., Yang X.-M. (2016). Inhibition of microRNA-126 promotes the expression of Spred1 to inhibit angiogenesis in hepatocellular carcinoma after transcatheter arterial chemoembolization: In vivo study. Onco Targets Ther..

[118] Beyer S., Fleming J., Meng W., Singh R., Haque S.J., Chakravarti A. (2017). The Role of miRNAs in angiogenesis, invasion and metabolism and their therapeutic implications in gliomas. Cancers.

[119] Markus R., Chow V., Pan Z., Hanes V. (2017). A phase I, randomized, single-dose study evaluating the pharmacokinetic equivalence of biosimilar ABP 215 and bevacizumab in healthy adult men. Cancer Chemother. Pharmacol..

[120] Hu-Lowe D.D., Zou H.Y., Grazzini M.L., Hallin M.E., Wickman G.R., Amundson K., Chen J.H., Rewolinski D.A., Yamazaki S., Wu E.Y. (2008). Nonclinical antiangiogenesis and antitumor activities of axitinib (AG-013736), an oral, potent, and selective inhibitor of vascular endothelial growth factor receptor tyrosine kinases 1, 2, 3. Clin. Cancer Res..

[121] McNamara M.G., Le L.W., Horgan A.M., Aspinall A., Burak K.W., Dhani N., Chen E., Sinaei M., Lo G., Kim T.K. (2015). A Phase II trial of second-line axitinib following prior antiangiogenic therapy in advanced hepatocellular carcinoma. Cancer.

[122] Sandler A., Gray R., Perry M.C., Brahmer J., Schiller J.H., Dowlati A., Lilenbaum R., Johnson D.H. (2006). Paclitaxel–carboplatin alone or with bevacizumab for non–small-cell lung cancer. N. Engl. J. Med..

[123] Hurwitz H., Fehrenbacher L., Novotny W., Cartwright T., Hainsworth J., Heim W., Berlin J., Baron A., Griffing S., Holmgren E. (2004). Bevacizumab plus irinotecan, fluorouracil, and leucovorin for metastatic colorectal cancer. N. Engl. J. Med..

[124] Sunwoo J.B., Chen Z., Dong G., Yeh N., Bancroft C.C., Sausville E., Adams J., Elliott P., Van Waes C. (2001). Novel proteasome inhibitor PS-341 inhibits activation of nuclear factor-κB, cell survival, tumor growth, and angiogenesis in squamous cell carcinoma. Clin. Cancer Res..

[125] Roccaro A.M., Hideshima T., Raje N., Kumar S., Ishitsuka K., Yasui H., Shiraishi N., Ribatti D., Nico B., Vacca A. (2006). Bortezomib mediates antiangiogenesis in multiple myeloma via direct and indirect effects on endothelial cells. Cancer Res..

[126] Corn P.G., Varkaris A., Li Ning Tapia E.M., Araujo J.C., Aparicio A., Tu S.-M., Zurita A.J., Efstathiou E., Qiao W., Wen S. (2013). Modulation of soluble c-Met, bone turnover markers, angiogenic factors, and c-Met in men with mCRPC treated with cabozantinib. Am. Soc. Clin. Oncol..

[127] Liu J., Barry W.T., Birrer M.J., Lee J.-M., Buckanovich R.J., Fleming G.F., Rimel B., Buss M.K., Nattam S.R., Hurteau J. (2014). A randomized phase 2 trial comparing efficacy of the combination of the PARP inhibitor olaparib and the antiangiogenic cediranib against olaparib alone in recurrent platinum-sensitive ovarian cancer. Am. Soc. Clin. Oncol..

[128] Schoumacher M., Burbridge M. (2017). Key roles of AXL and MER receptor tyrosine kinases in resistance to multiple anticancer therapies. Curr. Oncol. Rep..

[129] Seront E., Rottey S., Sautois B., Kerger J., D’hondt L., Verschaeve V., Canon J.-L., Dopchie C., Vandenbulcke J., Whenham N. (2012). Phase II study of everolimus in patients with locally advanced or metastatic transitional cell carcinoma of the urothelial tract: Clinical activity, molecular response, and biomarkers. Ann. Oncol..

[130] Quintás-Cardama A., Kantarjian H.M., Manshouri T., Thomas D., Cortes J., Ravandi F., Garcia-Manero G., Ferrajoli A., Bueso-Ramos C., Verstovsek S. (2009). Lenalidomide plus prednisone results in durable clinical, histopathologic, and molecular responses in patients with myelofibrosis. J. Clin. Oncol..

[131] Tefferi A., Cortes J., Verstovsek S., Mesa R.A., Thomas D., Lasho T.L., Hogan W.J., Litzow M.R., Allred J.B., Jones D. (2006). Lenalidomide therapy in myelofibrosis with myeloid metaplasia. Blood.

[132] Koyama N., Saito K., Nishioka Y., Yusa W., Yamamoto N., Yamada Y., Nokihara H., Koizumi F., Nishio K., Tamura T. (2014). Pharmacodynamic change in plasma angiogenic proteins: A dose-escalation phase 1 study of the multi-kinase inhibitor lenvatinib. BMC Cancer.

[133] Soria J.-C., DeBraud F., Bahleda R., Adamo B., Andre F., Dientsmann R., Delmonte A., Cereda R., Isaacson J., Litten J. (2014). Phase I/IIa study evaluating the safety, efficacy, pharmacokinetics, and pharmacodynamics of lucitanib in advanced solid tumors. Ann. Oncol..

[134] Liu J.F., Barry W.T., Birrer M.J., Lee J.-M., Buckanovich R.J., Fleming G.F., Rimel B., Buss M.K., Nattam S.R., Hurteau J. (2017). Overall survival and updated progression-free survival results from a randomized phase 2 trial comparing the combination of olaparib and cediranib against olaparib alone in recurrent platinum-sensitive ovarian cancer. Am. Soc. Clin. Oncol..

[135] Tran H.T., Liu Y., Zurita A.J., Lin Y., Baker-Neblett K.L., Martin A.-M., Figlin R.A., Hutson T.E., Sternberg C.N., Amado R.G. (2012). Prognostic or predictive plasma cytokines and angiogenic factors for patients treated with pazopanib for metastatic renal-cell cancer: A retrospective analysis of phase 2 and phase 3 trials. Lancet Oncol..

[136] Palani R., Apperley J.F., Reid A., Foroni L., Deplano S., Milojkovic D. (2015). Thyroid function abnormalities associated with ponatinib therapy in patients with chronic myeloid leukemia. Thyroid.

[137] Fuchs C.S., Tomasek J., Yong C.J., Dumitru F., Passalacqua R., Goswami C., Safran H., dos Santos L.V., Aprile G., Ferry D.R. (2014). Ramucirumab monotherapy for previously treated advanced gastric or gastro-oesophageal junction adenocarcinoma (REGARD): An international, randomised, multicentre, placebo-controlled, phase 3 trial. Lancet.

[138] Tamaskar I., Garcia J.A., Elson P., Wood L., Mekhail T., Dreicer R., Rini B.I., Bukowski R.M. (2008). Antitumor effects of sunitinib or sorafenib in patients with metastatic renal cell carcinoma who received prior antiangiogenic therapy. J. Urol..

[139] Fiedler W., Mesters R., Tinnefeld H., Loges S., Staib P., Dührsen U., Flasshove M., Ottmann O.G., Jung W., Cavalli F. (2003). A phase 2 clinical study of SU5416 in patients with refractory acute myeloid leukemia. Blood.

[140] Giles F.J., Stopeck A.T., Silverman L.R., Lancet J.E., Cooper M.A., Hannah A.L., Cherrington J.M., O'Farrell A.-M., Yuen H.A., Louie S.G. (2003). SU5416, a small molecule tyrosine kinase receptor inhibitor, has biologic activity in patients with refractory acute myeloid leukemia or myelodysplastic syndromes. Blood.

[141] Moroney J., Fu S., Moulder S.L., Falchook G.S., Helgason T., Levenback C.F., Hong D.S., Naing A., Wheler J.J., Kurzrock R. (2012). Phase I study of the anti-angiogenic antibody bevacizumab and the mTOR/hypoxia-inducible factor inhibitor temsirolimus combined with liposomal doxorubicin. Clin. Cancer Res..

[142] Mayer E.L., Isakoff S.J., Klement G., Downing S.R., Chen W.Y., Hannagan K., Gelman R., Winer E.P., Burstein H.J. (2012). Combination antiangiogenic therapy in advanced breast cancer: A phase 1 trial of vandetanib, a VEGFR inhibitor, and metronomic chemotherapy, with correlative platelet proteomics. Breast Cancer Res. Treat..

[143] Drevs J., Müller-Driver R., Wittig C., Fuxius S., Esser N., Hugenschmidt H., Konerding M.A., Allegrini P.R., Wood J., Hennig J. (2002). PTK787/ZK 222584, a specific vascular endothelial growth factor-receptor tyrosine kinase inhibitor, affects the anatomy of the tumor vascular bed and the functional vascular properties as detected by dynamic enhanced magnetic resonance imaging. Cancer Res..

[144] Chen H., Modiano M., Neal J., Brahmer J., Rigas J., Jotte R., Leighl N., Riess J., Kuo C., Liu L. (2014). A phase II multicentre study of ziv-aflibercept in combination with cisplatin and pemetrexed in patients with previously untreated advanced/metastatic non-squamous non-small cell lung cancer. Br. J. Cancer.

